# Effect of Redundant Haptic Information on Task Performance during Visuo-Tactile Task Interruption and Recovery

**DOI:** 10.3389/fpsyg.2016.01924

**Published:** 2016-12-08

**Authors:** Hee-Seung Moon, Jongsoo Baek, Jiwon Seo

**Affiliations:** ^1^School of Integrated Technology, Yonsei UniversityIncheon, Korea; ^2^Yonsei Institute of Convergence Technology, Yonsei UniversityIncheon, Korea

**Keywords:** task interruption and recovery, multitasking, multimodal task, working memory, haptic stimuli

## Abstract

Previous studies have revealed that interruption induces disruptive influences on the performance of cognitive tasks. While much research has focused on the use of multimodal channels to reduce the cost of interruption, few studies have utilized haptic information as more than an associative cue. In the present study, we utilized a multimodal task interruption scenario involving the simultaneous presentation of visual information and haptic stimuli in order to investigate how the combined stimuli affect performance on the primary task (cost of interruption). Participants were asked to perform a two-back visuo-tactile task, in which visual and haptic stimuli were presented simultaneously, which was interrupted by a secondary task that also utilized visual and haptic stimuli. Four experimental conditions were evaluated: (1) paired information (visual stimulus + paired haptic stimulus) with interruption; (2) paired information without interruption; (3) non-paired information (visual stimulus + non-paired haptic stimulus) with interruption; and (4) non-paired information without interruption. Our findings indicate that, within a visuo-tactile task environment, redundant haptic information may not only increase accuracy on the primary task but also reduce the cost of interruption in terms of accuracy. These results suggest a new way of understanding the task recovery process within a multimodal environment.

## Introduction

In daily life, people face various cognitive tasks, such as sending an e-mail or entering data into a computer in their workspace or home. Usually, these tasks are quite simple and completed with no errors. However, people often encounter circumstances in which another unexpected task interrupts the execution of the prior task. In practice, interruptions between multiple cognitive tasks occur frequently, and researchers have investigated these shifts in attention in workspaces via observational studies (Chisholm et al., 2000; Czerwinski et al., 2004; González and Mark, 2004). Numerous studies have attempted to identify how interruptions affect tasks and how people resume their original tasks after interruptions within a typical workspace (Czerwinski et al., 2004; Mark et al., 2005; Iqbal and Horvitz, 2007). For instance, a ring tone from a phone call, the arrival of a new e-mail, or a question from a colleague can all represent external interruptions that occur while performing a primary task that engages a person's attention (examples given by Fisher, 1998). With advanced technology, the number of complex situations and potential interruptions that divide people's attention has rapidly increased.

Recently, psychologists and human-computer interaction researchers have begun to focus on understanding the role of interruptions in cognitive control. A number of studies have revealed that interruptions are disruptive: Interruption by a secondary task causes interference in performing the primary task. Baddeley et al. (1984) demonstrated concurrent decreases in performance on two simultaneous tasks that require cognitive resources and therefore use working memory. Recent studies also focused on what makes interruptions disruptive, confirming this in two ways. Firstly, resuming the primary task requires more time following an interruption, a phenomenon referred to as resumption lag (Hodgetts and Jones, 2006; Monk et al., 2008; Brumby et al., 2013). In addition, interruptions lead to an increase in the likelihood of errors within the recovered task (Trafton et al., 2011; Brumby et al., 2013). These two prominent influences are common within different kinds of tasks, such as simple data-entry tasks (Zish and Trafton, 2014), sequential tasks (Trafton et al., 2011), cognitively demanding tasks (Borst et al., 2015), and decision-making tasks (Gathmann et al., 2015). Research in the field of human-computer interaction has also examined task switching and cognitive control in order to predict human task performance (Hornof et al., 2010). In addition, task switching has been noted for its effects on performance and mental load in both single-modal (Bailey et al., 2001) and multimodal user interfaces (Lu et al., 2013).

In order to make a precise prediction of performance on novel tasks, researchers have endeavored to elucidate the entire cognitive recovery process. Working memory is utilized for the maintenance and processing of information in the task at hand (Barrouillet et al., 2011) and is considered crucial for shifting cognitive tasks (Drews and Musters, 2015). Barrouillet et al. (2004) proposed a model of time-based resource management with regard to the maintenance and processing aspects of working memory. According to this model, information associated with the current task can undergo a decay process when attention toward the task is switched. In addition, task switching results in decreased recall performance (Liefooghe et al., 2008).

When people are faced with a situation in which their primary task is interrupted by a secondary task, information regarding the primary task is stored in working memory until resumption of the task following completion of the secondary task (Trafton et al., 2003). This ability to multitask is a common capability that allows most people to deal with interruptions without grave hardship. However, due to the limited capacity of working memory, the new information relevant to the secondary task can interfere with the information related to the primary task (Drews and Musters, 2015). The storage capacity of working memory has been researched for decades, and it is now well-known that the central capacity is limited to a few chunks of information at a time (Cowan, 2001). Beyond the central capacity (i.e., shared memory capacity for several modalities), Saults and Cowan (2007) revealed the capacity of the peripheral memory for specific modalities (e.g., visual or auditory modality). As each modality has its own peripheral resources, humans can recall more information when both central and peripheral memory systems of different modalities are involved (Cowan et al., 2014).

The Memory-For-Goals (MFG) theory represents one of the most popular frameworks for conveying the effect of interruptions (Altmann and Trafton, 2002). The MFG theory states that the interruption and recovery processes are based on the idea that human memory has a required activation level for each task and its associated goal. Like the working memory model proposed by Barrouillet et al. (2004), the MFG theory asserts that activation associated with a cognitive task decays over time (Altmann and Trafton, 2002). Borst et al. (2015) also specified the interruption and recovery processes in terms of information transference between the problem state and declarative memory. The problem state is a resource that stores requisite information for performing a cognitive task. When a primary task is interrupted, the existing information in the problem state moves to declarative memory, and novel information associated with the interrupting task becomes stored in the problem state. After transference to declarative memory, information associated with the primary task decays over time in terms of a power function (Borst et al., 2015). In addition, the interrupting task increases its own activation level, which produces increased interference on the primary task (Altmann and Trafton, 2002). Several studies have supported this MFG framework, revealing that, when participants are interrupted such that they are required to perform a longer task, increases in resumption lag and the number of errors are observed (Hodgetts and Jones, 2006; Monk et al., 2008; Brumby et al., 2013; Altmann et al., 2014; Borst et al., 2015).

A large proportion of studies have conducted simple visual tasks in laboratory environments using monitors, whereas relatively few studies have utilized a multi-sensory task environment. As real-world tasks occur under multi-sensory circumstances, the recovery process should be studied within multimodal task environment. Hodgetts et al. (2014) and Keus van de Poll and Sörqvist (2016) focused on the auditory modality and investigated the effects of auditory distraction on a visual task recovery. Hodgetts et al. (2014) implemented a command and control task interrupted by yes/no questionnaires with auditory noise. Keus van de Poll and Sörqvist (2016) utilized a writing task interrupted by arithmetic problems with background speech. The results of both studies indicated that the interruption recovery process in a visual modality is affected by distraction from an auditory modality.

Haptic sensation is another less-studied modality involved in multimodal interruption recovery processes. Haptic feedback has been applied in various fields such as remote surgery (Prattichizzo et al., 2012), in-car messaging (Ardoin and Ferris, 2016), and virtual reality (Corbett et al., 2016). For example, Corbett et al. (2016) demonstrated that haptic feedback enhances users' performance in a virtual pointing task. Nam et al. (2008) further revealed that realistic haptic feedback regarding the movement of the puck and stick improves performance during a virtual air hockey game. Furthermore, the presence of haptic feedback increases participant immersion in a virtual surgery environment (Meijden and Schijven, 2009). However, these studies do not explain the effects of redundant haptic information during multimodal task interruption and recovery. As haptic information is always perceived naturally during our daily cognitive tasks, it is important to understand the precise cognitive processes underlying the influence of this complex modality.

Studies of multimodal task interruption have utilized haptic sensation as an associative cue in order to enhance the activation of the primary task (Hopp et al., 2005; Prewett et al., 2012). When an associative cue and a primary task occur simultaneously, a link between the two is generated, allowing activation of the primary task following the presentation of the associative cue (Altmann and Trafton, 2002). Several studies have therefore applied associated cues in order to increase performance during multitasking (Altmann and Trafton, 2004; Hopp et al., 2005; Hodgetts and Jones, 2006; Smith et al., 2009). Furthermore, Prewett et al. (2012) demonstrated that using a vibrotactile cue (which is obviously non-visual) as an alert or message is more effective than using a visual cue when the primary task is visual. The multiple resource theory (Wickens, 2002) supports the effectiveness of vibrotactile cueing in this multitasking scenario. Wickens (2002) suggested that attentional resources from a separate resource pool distinguished by different sensory modalities can be successfully divided in parallel. Within the framework of the multiple resource theory, Hopp et al. (2005) also suggested that vibrotactile cues help alleviate the cost of interruption by reducing reaction time when the primary decision making task is visual.

However, previous studies utilizing simple vibration motors to implement vibrotactile cues have a clear limitation in that only directional or spatial cue information may be provided (Prewett et al., 2012). Thus, more general haptic sensations beyond vibrotactile cueing should be investigated, particularly in multimodal situations.

Though few in number, some studies have indeed utilized dual task situations that included haptic stimuli. Lu et al. (2013) performed a meta-analysis of studies regarding multimodal dual task situations in which a primary visual task was interrupted by secondary tasks of various modalities, including a haptic modality. As previously noted, interruption of a primary visual task with a secondary haptic task resulted in increased performance relative to interruption of a primary visual task with a secondary visual task. The multiple resource theory proposed by Wickens (2002) may account for such a result. However, in real-world situations, both the primary and secondary tasks rely on multiple modalities. Among various possible combinations of multiple modalities, we aimed to focus on the combination of visual and haptic modalities (visuo-tactile primary task + visuo-tactile secondary task).

In the present study, we implemented a combined visuo-tactile task in order to investigate the effect of redundant haptic stimuli during a task interruption situation. We first investigated the effect of redundant haptic information on the primary task. Our results align with those obtained by Lu et al. (2013), who studied the effect of redundant auditory information, which has been shown to increase accuracy as well as reaction time during task performance. We then studied the role of haptic stimuli in the interruption recovery process. Specifically, we analyzed how the combined information from paired visual and haptic modalities affects the recovery process relative to non-paired visual and haptic information. Based on the MFG theory, we speculate that priming from the redundant haptic stimulus may exert beneficial effects on the task recovery process. In this paper, we define “task recovery” as the retrieving process of a primary task information after the primary task is distracted by an interrupting task. As few studies have examined this topic, we addressed the following research questions:

- Q1: Does the cost of task interruption fit the MFG theory in a visuo-tactile task environment?
- Q2: How does the presence of redundant haptic information affect performance in a visuo-tactile cognitive task?
- Q3: Is there any benefit of using redundant haptic information, especially in a visuo-tactile task interruption and recovery process?

## Materials and methods

Our experiment was characterized by a 2 (Interruption: present vs. absent) × 2 (Haptic Information: paired vs. non-paired) within-subject factorial design. Therefore, four experimental conditions were used: the two-back visuo-tactile task with paired haptic stimuli (H_p_), with and without interruption, and the same two-back task with non-paired haptic stimuli (H_n_), also with and without interruption. To create a visuo-tactile task environment that included haptic stimuli, a seven degree-of-freedom haptic device was used in conjunction with a PC, monitor, and keyboard.

### Participant

Twenty-one students from Yonsei University (14 men; 7 women; age range: 20–26 years, mean age: 22.1 ± 2.36 years) participated in the present study. All participants were right-handed, with no visual or manual impairments, and remained naïve to the purpose of the experiments. No participant had any previous experience with relevant task interruption and recovery experiments. Participants conducted the procedure in a laboratory environment with one experimenter. All individual sessions lasted approximately 1 h. The present study was performed in accordance with the ethical standards laid down by the 1964 Declaration of Helsinki. All study participants provided informed written consent. Following the relevant Act and Enforcement Rules, which are specified below, from the Korean Ministry of Health and Welfare, our experimental procedure is exempt from local ethics committee approval. According to Article 15 (2) of the Bioethics and Safety Act and Article 13 of the Enforcement Rule of Bioethics and Safety Act, a research project “which utilizes a measurement equipment with simple physical contact that does not cause any physical change in the subject” (Korean to English translation by the authors) is exempt from such approval. Our entire experimental procedure was designed to use only a PC and a haptic device that does not cause any physical change in the participant.

### Materials

**Figure 1:**
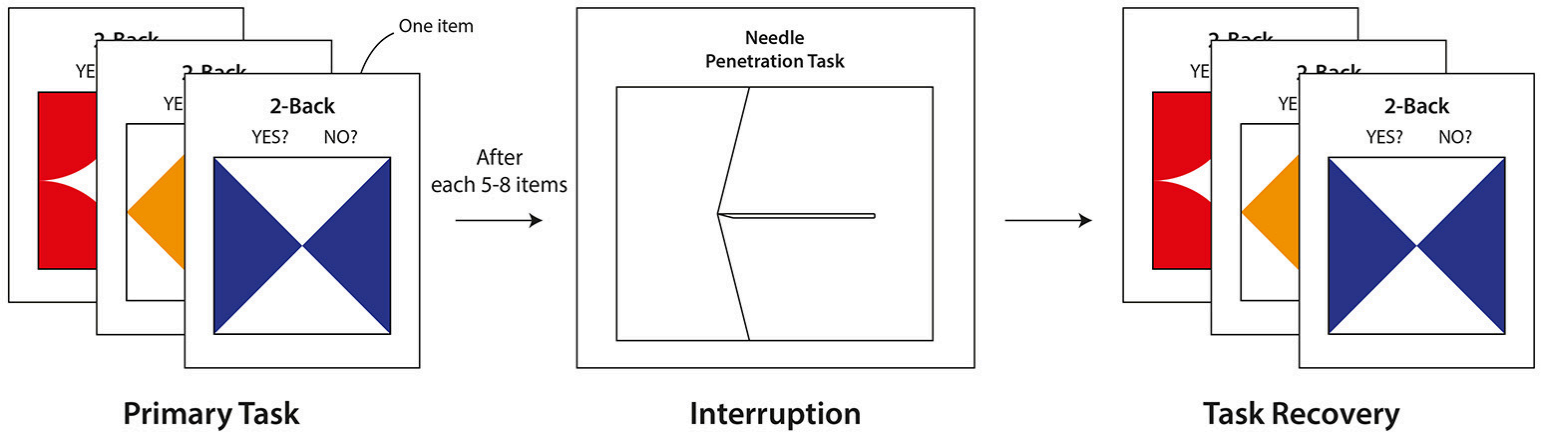
**Visuo-tactile two-back task interrupted by virtual needle penetration task**.

**Figure 2:**
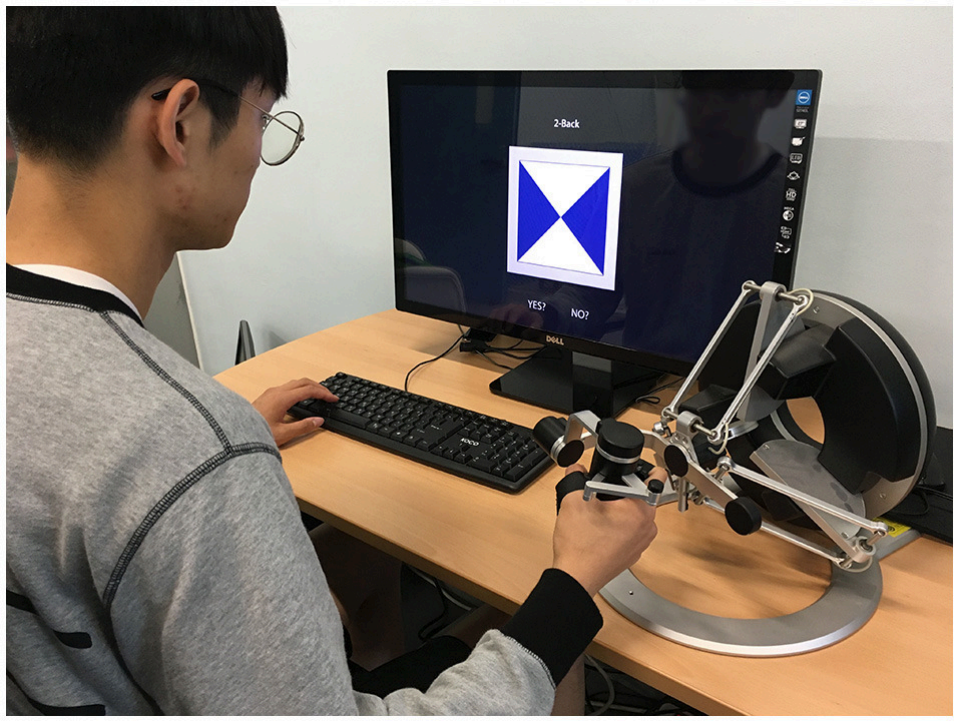
**Experimental setup with a haptic device and a PC**.

Two kinds of cognitive tasks were used in the present study: a two-back visuo-tactile task and a virtual needle penetration task (Figure 1). Each task was implemented as the primary task and the interrupting task. The primary task (two-back visuo-tactile task) is based upon the N-back design, which is widely utilized for the measurement of working memory in cognitive neuroscience (Kane et al., 2007). Furthermore, this cognitively demanding task has been used to assess the effects of task interruption (Monk et al., 2008; Borst et al., 2015). In order to provide participants with a visuo-tactile experimental environment including precise haptic feedback, our experimental system was composed of one haptic interface (Omega.7, a highly precise force-feedback haptic device with seven degrees-of-freedom produced by Force Dimension, Switzerland) and one PC (Figure 2). A 27-inch display monitor and a keyboard were set up in front of the participant. The haptic device was placed near the dominant hand; because all participants were right-handed, the haptic device was positioned on the right side of the monitor. The haptic interface can be connected and accessed through the Haptik Library (De Pascale and Prattichizzo, 2007). In addition, both haptic tasks were developed based on CHAI3D, an open-source set of C++ libraries for real-time haptic simulation, and driven with the Windows 8.1 operating system.

#### Two-back visuo-tactile task (primary task)

Participants were asked to perform the two-back visuo-tactile task as the primary task. A stream of visuo-tactile stimuli (cards with visual images and haptic stimuli) were presented sequentially, and participants were required to determine whether the information on the current item was the same as that occurring two items before. Hence, participants were required to keep this information in their working memory while recognizing new information.

**Figure 3:**
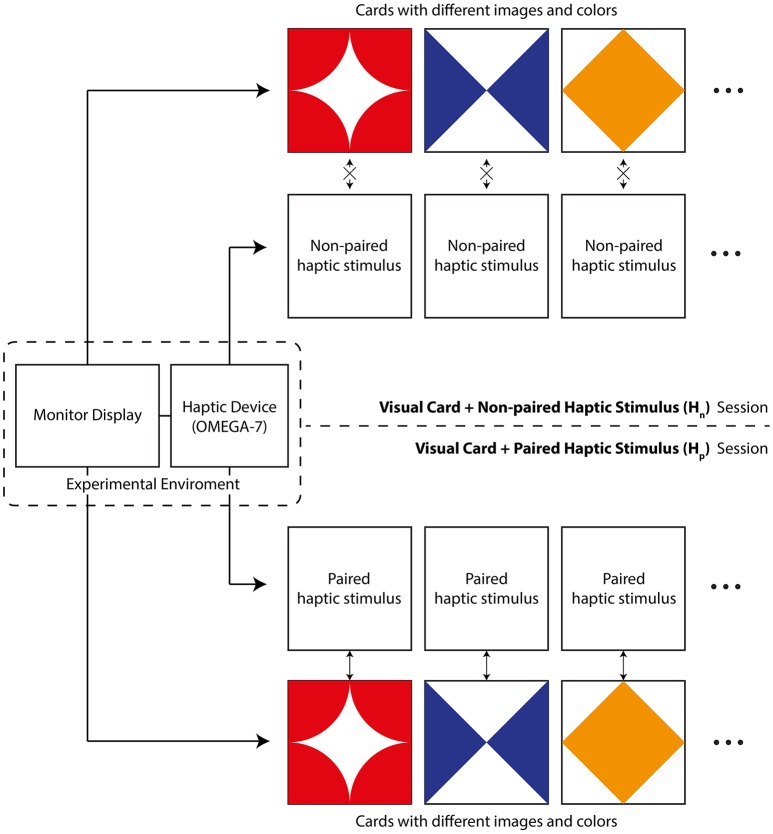
**Overall schematic of the visuo-tactile two-back task**.

In total, nine visuo-tactile stimuli (nine visual cards and nine tactile stimuli occurring in conjunction with one another) were used in the primary task. Since we presented visual and haptic stimuli to a participant at the same time, the information given to the participant was divided into two channels. Figure 3 shows an overall schematic diagram of the two-back visuo-tactile task. First, visual information was provided via a 27-inch monitor as a series of rectangular cards, measuring 5 in × 5 in (Figure 3). All nine visual cards were distinguishable as nine different images with nine different colors. A haptic device provided haptic stimuli paired with the aforementioned visual cards. Nine haptic stimuli were generated using CHAI3D and the haptic simulation library as follows. Note that these stimuli are more general than the vibrotactile stimulus utilized in previous studies.

- Viscosity: high-viscosity, mid-viscosity, low-viscosity
- Stiffness: high-stiffness, mid-stiffness, low-stiffness
- Vibration: strong-vibration, mid-vibration, weak-vibration

The different visual cards and haptic stimuli were paired with one another and simultaneously presented to the participant in the H_p_ session. Each paired item was presented for 2400 ms, followed by a mask of 240 ms. Participants responded to each item by pressing the corresponding key on the keyboard for each answer: “1” or “y” for “Yes” (i.e., the current item is the same as the item that occurred two items before) and “2” or “n” for “No.” For participants who were uncomfortable with pressing two distant keys (“y” and “n”), two nearby keys (“1” and “2”) were also offered as an alternative option. Each participant chose and used one option (i.e., “y”/“n” or “1”/“2”) depending on his or her preference throughout the whole experiment. If a participant did not respond within the given time (2400 ms), the response was recorded as a failure (i.e., wrong answer).

Two experimental sessions were implemented in order to investigate the effects of paired haptic information: a paired haptic stimulus (H_p_) session and a non-paired haptic stimulus (H_n_) session. In the H_n_ session, participants were presented with a non-paired haptic stimulus and a visual card. The non-paired haptic stimulus was arbitrarily chosen for each visual card. Both sessions of the primary task were interrupted every five to eight items (randomly assigned). Each session consisted of five sets, and one set consisted of 60 items.

#### Virtual needle penetration task (interrupting task)

As an interrupting task, a virtual needle penetration task, which demands cognitive resources from both visual and haptic senses, was implemented. This interrupting task was adapted from a needle insertion simulation toward haptic-rendered soft tissue originally designed by Gerovich et al. (2004) and Prattichizzo et al. (2012).

**Figure 4:**
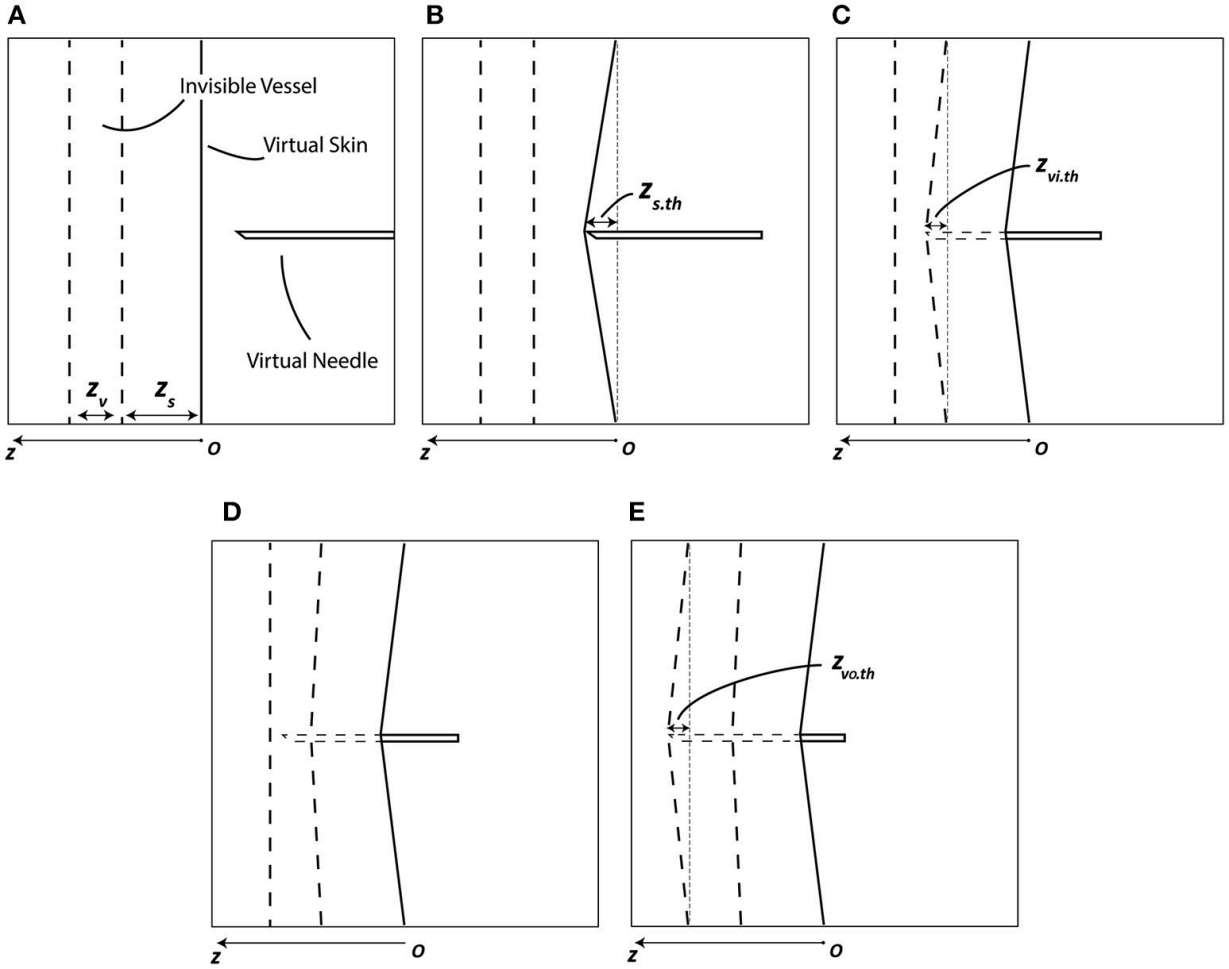
**Virtual needle penetration task**. The dashed lines in the figure indicate the invisible vessel walls and the invisible part of the needle. The solid lines indicate the visible skin and the visible part of the needle. **(A)** Before contact. **(B)** Needle penetrates the skin when *z* > *z*_*s*.*th*_. **(C)** Needle penetrates the vessel inward when *z* > *z*_*s*_ + *z*_*vi*.*th*_. **(D)** After successful penetration of vessel. **(E)** Needle penetrates the vessel outward when *z* > *z*_*s*_ + *z*_*v*_ + *z*_*vo*.*th*_; outward penetration indicates task failure.

A participant was instructed to move a virtual needle on the screen using the haptic device, find an invisible vessel inside the visible virtual skin, and place the tip of the needle inside the vessel (Figure 4). Throughout the interrupting task, the participant used only the right hand to manipulate the haptic device to control the virtual needle and also receive force feedback from the haptic device. The force feedback closely simulates the haptic sensation corresponding to the actual act of touching. The location of the invisible vessel was randomly assigned at each trial; however, the intensity of the force feedback at the moment penetrating the vessel was identical. Since the participant had become familiar with the force feedback intensity upon vessel penetration in the training period conducted prior to the actual experiment, the participant with proper concentration could successfully locate the needle inside the vessel. Given a 7200-ms time limit for the interrupting task, a participant was required to use his or her visual sense to penetrate the visible skin and haptic sense to locate the needle inside the vessel. The vessel had a certain thickness, z_v_, and the needle would pass through the other side of the vessel if the participant applied excessive force. When the participant held the tip of the needle inside the vessel for more than 1000 ms without passing through the vessel, the interrupting task successfully terminated.

Adapted from the simulation designed by Gerovich et al. (2004) and Prattichizzo et al. (2012), the following haptic renderings are implemented in this task. Three kinds of soft tissue were generated as virtual renderings of the skin, inward vessel wall, and outward vessel wall. Each layer was assigned distinct spring and damping coefficients. Therefore, participants could be provided haptic feedback such as spring stiffness during contact with the tissue as well as damping effect when the needle passed through any kind of tissue. A detailed haptic model was implemented as follows (based on the haptic model from Gerovich et al. (2004) and Prattichizzo et al. (2012), but simplified by removing some layers and viscous effects).

When the needle contacts and punctures the outermost layer (i.e., virtual skin):

(1) $$\begin{array}{l} F=k_{s}z,\;0<z<z_{s.th}\\ F=b_{s}zv,\;z_{s.th}<z<z_{s} \end{array}$$

When the needle interacts with the vessel wall, inward-bound:

(2) $$\begin{array}{l} F=k_{vi}\left(z-z_{s}\right)+b_{s}z_{s}v,\;z_{s}<z<z_{s}+z_{vi.th}\\ F=\left(b_{vi}z+b_{s}z_{s}\right)v,\;z_{s}+z_{vi.th}<z<z_{s}+z_{v} \end{array}$$

When the needle interacts with the vessel wall, outward-bound:

(3) $$\begin{array}{l} F=k_{vo}\left(z-z_{v}-z_{s}\right)+\left(b_{vi}z_{v}+b_{s}z_{s}\right)v,\;z_{s}+z_{v}<z<z_{s}+z_{v}\\ +\;z_{vo.th}\\ F=\left(b_{vo}z+b_{vi}z_{v}+b_{s}z_{s}\right)v,\;z_{s}+z_{v}+z_{vo.th}<z \end{array}$$

where *k*_*s*_, *k*_*vi*_, and *k*_*vo*_ represent the spring coefficients of corresponding tissues; *b*_*s*_, *b*_*vi*_, and *b*_*vo*_ represent the damping coefficients of corresponding layers (per unit thickness); *z*_*s*_ and *z*_*v*_ represent the thickness of the outer skin and vessel layers, respectively; and *z*_*s*.*th*_, *z*_*vi*.*th*_, and *z*_*vo*.*th*_ represent the thresholds for penetration shown in Figure 4.

In the interrupting task, the haptic modality was mainly used to determine the vessel's location. Meanwhile, the visual modality was used to monitor the movement of the virtual needle and confirm whether the skin had been penetrated. Unlike in the primary two-back task, the interrupting task was identical in the H_p_ and H_n_ sessions.

### Procedure

Each individual 1-h session was conducted in a laboratory environment. All participants were given a tutorial regarding the experimental procedures, including a clear explanation of the tasks. Prior to the actual experiment, participants engaged in a training session in order to familiarize them with the haptic interface and experimental tasks. The primary two-back task during the training period consisted of 40 H_p_ (paired haptic stimulus) items and 40 H_n_ (non-paired haptic stimulus) items. The goal of the training period was to ensure that participants had become accustomed to the nine visuo-tactile stimulus pairs of the primary two-back task and the force feedback intensity upon vessel penetration during the interrupting task.

After the training period, the actual experiment was conducted. Participants performed five sets of 60 items in each H_p_ and H_n_ session. Therefore, the entire experiment consisted of 10 sets per person. After every two sets, a participant was given a 3-min break. In order to reduce the potential effect due the order of the tasks, participants were equally divided into two groups; one performed the H_p_ session prior to the H_n_ session, while the other conducted the H_n_ session prior to the H_p_ session.

### Measures

To measure the effects of task interruption in a combined visuo-tactile task environment, we examined reaction time and accuracy as dependent variables. As previously mentioned, increased reaction time and decreased accuracy for the primary task have been highlighted as the major cost of task interruption (Hodgetts and Jones, 2006; Monk et al., 2008; Trafton et al., 2011; Brumby et al., 2013). For every primary two-back task item, the time interval between the moment when a participant received the visuo-tactile stimulus and the moment when the participant pressed the response key was recorded as the reaction time. Each H_p_ or H_n_ session consisted of five sets of 60 primary two-back task items, and the average reaction time of each participant for each session was measured. In addition, accuracy was also measured by recording the proportion of correct responses, and the average accuracy of each participant was also recorded for statistical analysis.

**Figure 5:**
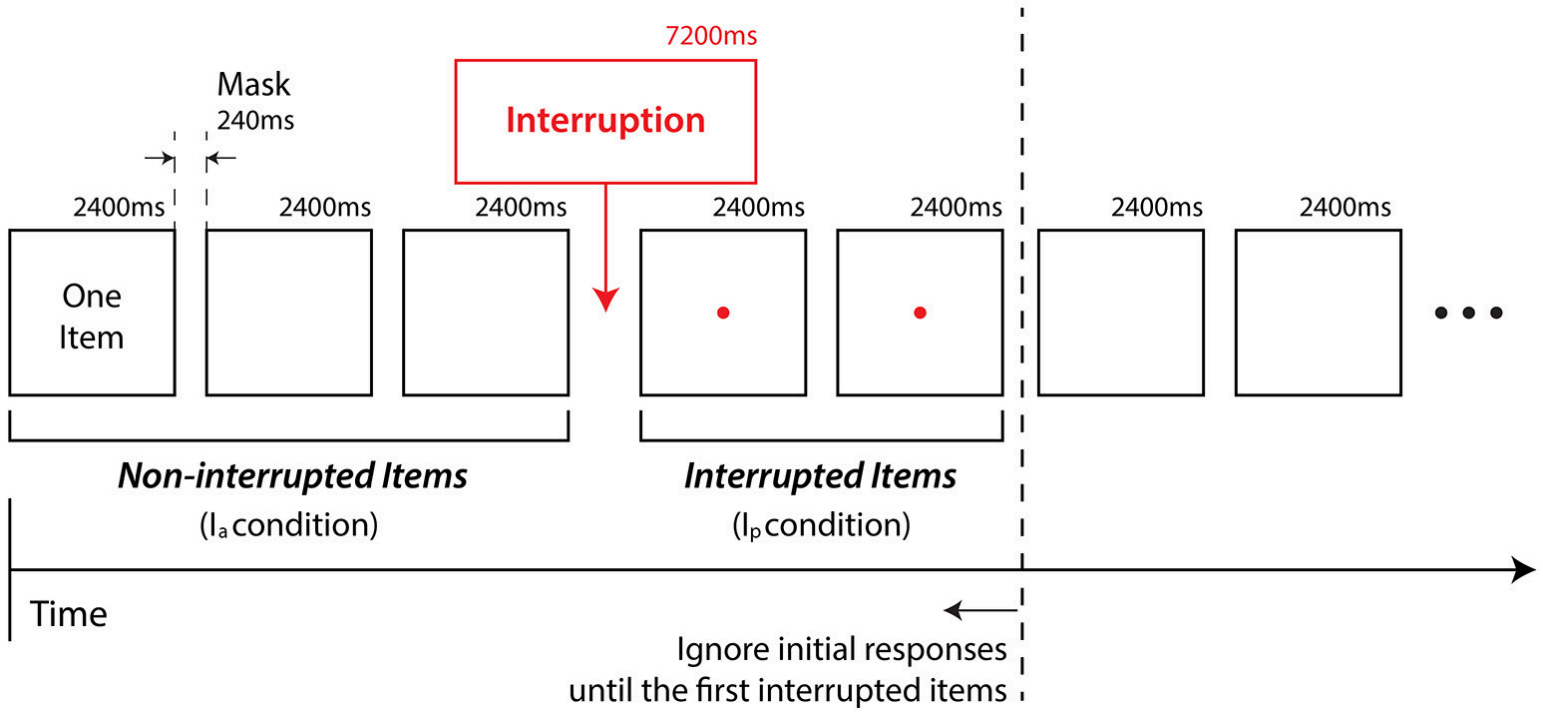
**Example sequence of two-back task items with interruption**.

Reaction time and accuracy were measured under four conditions: paired haptic stimulus with interruption present (H_p_ + I_p_), paired haptic stimulus with interruption absent (H_p_ + I_a_), non-paired haptic stimulus with interruption present (H_n_ + I_p_), and non-paired haptic stimulus with interruption absent (H_n_ + I_a_). Figure 5 depicts an example sequence of the task items and an interruption. Interruptions occurred at arbitrary points in the sequence. The next two items after an interruption were classified as interrupted items, while other items were classified as non-interrupted items. The performance of interrupted items was recorded as the condition with interruption present (I_p_). On the other hand, the performance of non-interrupted items was recorded as the condition with interruption absent (I_a_). Since initial responses can be extreme outliers (Borst et al., 2015) we also excluded the initial responses until the first interrupted items.

## Results

The cost of interruption recovery can be measured in two ways: reaction time and accuracy. As per the MFG theory, performance of the interrupted task would be degraded in terms of both reaction time and accuracy relative to the non-interrupted task (Altmann and Trafton, 2002; Monk et al., 2008; Altmann et al., 2014). In the present study, we investigated the effects of paired haptic stimulus presentation during interruption recovery. Thus, we first examined whether the haptic stimulus affected the performance of the primary task differently depending on the presence of interruption. Significant interactions between haptic stimulus presentation and the presence of an interruption with regard to accuracy or reaction time indicate that the haptic stimulus influences the interruption recovery process. As we observed these effects in our analysis, we further analyzed the effects of the haptic stimulus on accuracy. However, we observed no significant interaction between presentation of the haptic stimulus and the presence of interruption with regard to reaction time.

### Interactions between haptic stimulus presentation and the presence of interruption on reaction time and accuracy

The interaction between presentation of a haptic stimulus and the presence of interruption can be simply analyzed by examining the haptic benefit depending on the presence of interruption. In the present study, the haptic benefit was defined as an improvement in reaction time or accuracy due to the presence of the paired haptic stimulus (i.e., reaction time or accuracy under H_p_ condition minus reaction time or accuracy under H_n_ condition). Our analysis based on the subtracted data under two conditions is similar to the approach of Olesen et al. (2004).

We used a paired samples *t*-test to analyze the haptic benefit depending on the presence of interruption. The haptic benefit in terms of reaction time under the interrupted condition was similar to the haptic benefit under the non-interrupted condition (*t* = 1.526, *p* = 0.143, Cohen's *d* = 0.333, according to the 5-percent-standard level; non-interrupted task mean = 220.43, *SE* = 31.11; interrupted task mean = 133.38, *SE* = 51.69). Thus, we observed no significant interaction between presentation of the haptic stimulus and the presence of interruption with regard to reaction time. In contrast, the haptic benefit in terms of accuracy under the interrupted condition was significantly better than the haptic benefit under the non-interrupted condition (*t* = −5.640, *p* < 0.01, Cohen's *d* = 1.231, according to the 5-percent-standard level; non-interrupted task mean = 0.70, *SE* = 0.68; interrupted task mean = 8.03, *SE* = 1.32). Thus, we observed a significant interaction between the presentation of the haptic stimulus and the presence of interruption with regard to accuracy. The same result was obtained by the two-way repeated measures analysis of variance (ANOVA) described in the next section.

### Effect of haptic stimulus on accuracy during interruption recovery process

We measured the accuracy of participants in the primary two-back task under the aforementioned four conditions (i.e., H_p_ + I_p_, H_p_ + I_a_, H_n_ + I_p_, H_n_ + I_a_). Mean accuracy values for the 21 included participants are presented in Table 1. As we predicted based on the MFG theory, participants made more errors in the interrupted condition than in the non-interrupted condition (90.18 vs. 71.23%, on average). Two-way repeated measures ANOVA were used to discover significant differences among each condition. The presence of paired haptic stimuli and the presence of interruption were used as within-subject factors in the two-way repeated measures ANOVA.

**Table 1:** **Accuracy in the primary two-back task**.

**Condition**	**Range (%)**	**Mean (%)**	***SD* (%)**	**Combined Mean (%)**
H_p_+I_p_ (Paired Haptic Stimulus, Interrupted)	80.6–97.0	90.47	4.58	90.18
H_n_+I_p_ (Non-paired Haptic Stimulus, Interrupted)	77.4–97.0	89.80	5.83	
H_p_+I_a_ (Paired Haptic Stimulus, Non-interrupted)	60.1–88.0	75.17	6.78	71.23
H_n_+I_a_ (Non-paired Haptic Stimulus, Non-interrupted)	48.0–76.0	67.23	8.02	

**Figure 6:**
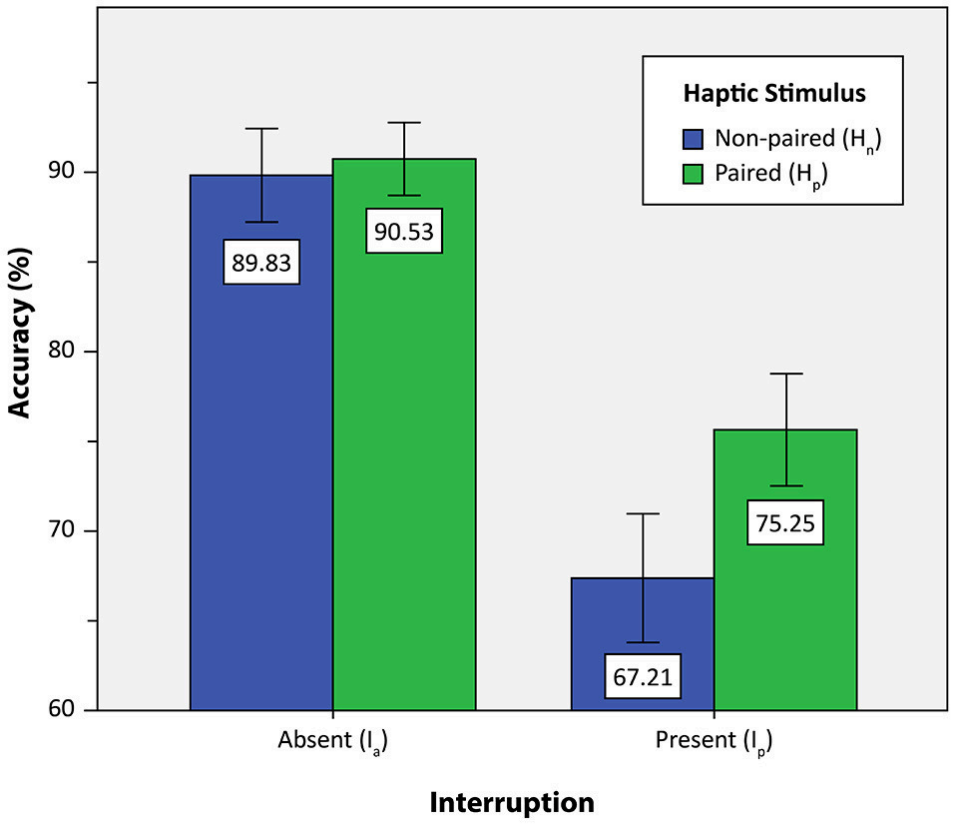
**Measured accuracy under the four conditions**. Error bars represent the double standard error of the mean.

Figure 6 depicts participant accuracy under the four conditions of the present study. When participants were interrupted by the interrupting task, their accuracy on the primary two-back task decreased significantly [*F*_(1, 20)_ = 237.100, MSE = 31.801, *p* < 0.01, η^2^ = 0.922]. In addition, participants achieved significantly better accuracy when paired haptic stimuli were provided than when non-paired haptic stimuli were provided [*F*_(1, 20)_ = 28.122, MSE = 14.254, *p* < 0.01, η^2^ = 0.584]. Furthermore, we observed a significant interaction between the presentation of a haptic stimulus and the presence of interruption with regard to accuracy, in accordance with our *t*-test results discussed in the previous section [*F*_(1, 20)_ = 31.815, MSE = 8.870, *p* < 0.01, η^2^ = 0.614]. That is, the paired haptic stimulus further enhances accuracy when the task is interrupted relative to when the task is not interrupted.

Our data analysis confirms the following results regarding the effect of paired haptic stimuli on task performance during visuo-tactile task interruption and the subsequent recovery process: (1) The presence of a paired haptic stimulus improves accuracy on the primary two-back task; (2) the paired haptic stimulus affects the accuracy differently depending on the presence of interruption; the paired haptic stimulus further enhances the accuracy when the interruption is present.

## Discussion

Multiple sensory channels have been studied to understand the cognitive processes involved in multimodal task interruption and recovery. Previous studies have focused on visual and auditory modalities in particular, while studies involving haptic stimuli have been limited to an investigation of their role as associative cues in enhancing performance during the primary task. In the present study, we conducted a visuo-tactile task interruption and recovery experiment in order to examine the role of redundant haptic information in such processes. Our results confirm that the use of redundant haptic information enhances participant accuracy on the primary visuo-tactile task. Noticeably, the redundant haptic information is especially helpful for participants to recover the interrupted task. This increase in accuracy is significantly greater during interruption recovery than during a non-interrupted task.

- Q1: Does the cost of task interruption fit the MFG theory in a visuo-tactile task environment?

Our results align with the MFG theory, revealing an increased cost of task interruption with regard to accuracy. Regardless of the haptic stimulus, participants demonstrated decreased accuracy when they were interrupted. Hence, our data support the results of previous studies regarding interruption in a multimodal task environment (Hodgetts et al., 2014; Keus van de Poll and Sörqvist, 2016).

- Q2: How does the presence of redundant haptic information affect performance in a visuo-tactile cognitive task?

Our results indicate that redundant haptic information provided in the form of a paired haptic stimulus induces improvements in accuracy regardless of the presence of interruption. As mentioned earlier, Lu et al. (2013) investigated the use of redundant auditory information, which resulted in improved accuracy on a single visuo-auditory task without interruption. Our results indicated that similar enhancements are observed when redundant haptic information is provided during a visuo-tactile task.

- Q3: Is there any benefit of using redundant haptic information, especially in a visuo-tactile task interruption and recovery process?

The presence of the paired haptic stimulus resulted in significantly greater improvements in accuracy in the interrupted condition than in the non-interrupted condition, a comparison not studied in Lu et al. (2013). Such a result demonstrates that redundant haptic information exerts a specific influence on the interruption recovery process.

Similar to performance enhancements observed when associative cues are presented during the recovery process, increases in accuracy due to the presentation of redundant haptic information may be explained as a result of enhanced activation of the primary task, according to the MFG theory (Altmann and Trafton, 2002). An associative cue boosts the activation of the primary task (i.e., priming occurs) due to generation of a link between the cue and the primary task. Likewise, redundant haptic information can boost the activation of the primary task during the recovery process because a similar link between the haptic information and the primary task is generated.

The results of the present study suggest that haptic information may be effective for interruption management. In their meta-analysis, Lu et al. (2013) suggested various ways of using multimodal information for designing efficient multimodal interfaces. For example, haptic modalities can be effectively utilized to deliver low-complexity information such as simple notifications, while auditory modalities can be used to deliver high-complexity information such as informative alerts. However, such suggestions are based on limited experiments that have utilized a vibrotactile motor unable of delivering a complex haptic stimulus. However, the highly precise force feedback haptic device used in the present study is capable of generating highly complex haptic stimuli that can vary in terms of viscosity, stiffness, vibration, magnetic force, various textures, etc. The significant improvements in accuracy observed during the present study demonstrate the ability of haptic stimuli to provide such highly complex information for the management of interruptions. Our results may provide a foundation for elucidating the mechanisms underlying the recovery process in a multimodal sensory environment.

## Author contributions

HM and JS designed the study. HM developed the experimental software and performed the experiment. HM, JB, and JS analyzed the data and discussed the results. HM drafted the manuscript, and JB and JS revised the manuscript. All authors approved the final manuscript.

## Funding

This research was supported by the Ministry of Science, ICT, and Future Planning (MSIP), Korea, under the “IT Consilience Creative Program” (IITP-R0346-16-1008) and supervised by the Institute for Information & Communications Technology Promotion (IITP).

### Conflict of interest statement

The authors declare that the research was conducted in the absence of any commercial or financial relationships that could be construed as a potential conflict of interest.

## Abbreviations

ANOVA: Analysis of variance
MFG: Memory-For-Goals
Hp: paired haptic stimuli
Hn: non-paired haptic stimuli
Ip: interruption present
Ia: interruption absent.
